# Characterizing the Flavor Precursors and Liberation Mechanisms of Various Dry-Aging Methods in Cull Beef Loins Using Metabolomics and Microbiome Approaches

**DOI:** 10.3390/metabo12060472

**Published:** 2022-05-24

**Authors:** Derico Setyabrata, Kelly Vierck, Tessa R. Sheets, Jerrad F. Legako, Bruce R. Cooper, Timothy A. Johnson, Yuan H. Brad Kim

**Affiliations:** 1 Department of Animal Science, Purdue University, West Lafayette, IN 47907, USA or dericos@uark.edu (D.S.); sheets27@purdue.edu (T.R.S.); john2185@purdue.edu (T.A.J.); 2 Department of Animal and Food Science, Texas Tech University, Lubbock, TX 79409, USA or vierck@uark.edu (K.V.); jerrad.legako@ttu.edu (J.F.L.); 3 Bindley Bioscience Center, Purdue University, West Lafayette, IN 47907, USA; brcooper@purdue.edu

**Keywords:** dry-aging, cull cow, microbiome, amino acids, reducing sugars, metabolomics, volatile compounds

## Abstract

The objective of this study was to characterize and compare the dry-aging flavor precursors and their liberation mechanisms in beef aged with different methods. Thirteen paired loins were collected at 5 days postmortem, divided into four sections, and randomly assigned into four aging methods (wet-aging (WA), conventional dry-aging (DA), dry-aging in a water-permeable bag (DWA), and UV-light dry-aging (UDA)). All sections were aged for 28 days at 2 °C, 65% RH, and a 0.8 m/s airflow before trimming and sample collection for chemical, metabolomics, and microbiome analyses. Higher concentrations of free amino acids and reducing sugars were observed in all dry-aging samples (*p* < 0.05). Similarly, metabolomics revealed greater short-chain peptides in the dry-aged beef (*p* < 0.05). The DWA samples had an increase in polyunsaturated free fatty acids (C18:2trans, C18:3n3, C20:2, and C20:5; *p* < 0.05) along with higher volatile compound concentrations compared to other aging methods (aldehyde, nonanal, octanal, octanol, and carbon disulfide; *p* < 0.05). Microbiome profiling identified a clear separation in beta diversity between dry and wet aging methods. The *Pseudomonas* spp. are the most prominent bacterial species in dry-aged meat, potentially contributing to the greater accumulation of flavor precursor concentrations in addition to the dehydration process during the dry-aging. Minor microbial species involvement, such as *Bacillus* spp., could potentially liberate unique and potent flavor precursors.

## 1. Introduction

Dry-aging is a traditional aging method that has recently regained interest from both value-seeking consumers and niche market meat purveyors [1]. Unlike wet-aging (where meat is aged by storing in vacuum-packaged bags), the dry-aging process exposes the meat to a highly controlled environment without any protective packaging materials. These particular aging conditions have been associated with the development of unique flavors such as “beefy”, “buttery”, “nutty”, and “brown-roasted”, making the final product more flavorful [2]. However, the exact mechanism by which the dry-aging process could develop unique flavors or liberate flavor-related compounds has not been fully established. Furthermore, different dry-aging practices, such as the utilization of moisture-permeable bags and/or ultraviolet light, have been recently developed and could potentially alter the dry-aged flavor compound liberation process. Thus, the identification of the flavor precursor composition, which is integral to the dry-aging process, could provide beneficial information and practical insights to produce dry-aged beef products with consistent eating quality attributes.

In recent years, advancements in high-throughput analyses, such as metabolomics, have been adopted to determine changes in multiple biological systems, including the biochemical changes in meat products. Metabolomics analysis allows the profiling of small compounds (metabolites), elucidating the molecular changes responsible for meat quality development [3,4,5]. While limited, the utilization of metabolomics analysis has also been employed to identify compounds related to the meat flavor changes, showing increased concentration in small molecular flavor precursors (e.g., amino acids, nucleotides, vitamins, acids, and minerals) or decreased off-flavor related metabolites (e.g., terpenoids) in dry-aged beef products [2,6]. These studies exhibited the great potential of metabolomics analysis for use in profiling the flavor precursor composition of dry-aged meat products and elucidating the underlying mechanisms for the flavor development process.

Additionally, microbial activity during the dry-aging process has been suggested to contribute to flavor precursor liberation. For example, a previous study by Lee et al. [7] revealed that the greater presence of *Pilaira anomala* and *Debaryomyces hansenii* during dry-aging led to a greater abundance of free amino acids in the products. Those authors suggested that the microorganisms potentially released exogenous proteolytic and lipolytic enzymes, accelerating muscle breakdown during aging. However, the presence of various microbial species could potentially affect the flavor precursor development differently during the aging process. Therefore, characterization of the microbial community may be crucial to understanding the role of microbial presence and growth in the release of flavor precursors.

Thus, the main objective of this study was to characterize and compare the flavor precursors and the liberation mechanisms in beef aged under different aging conditions. In the current study, we utilized untargeted metabolomics and microbiome analyses coupled with multiple targeted chemical analyses, such as free amino acids, fatty acids, sugars, and volatile compounds contents to provide a comprehensive understanding of flavor precursor development after the application of aging. This study was a further elaboration of our previously published study, where significant changes in sensory palatability attributes were reported following the application of different aging methods (wet-aging (WA), conventional dry-aging (DA), dry-aging in a water-permeable bag (DWA), and UV-light dry-aging (UDA)) in cull cow beef loins [8].

## 2. Results

### 2.1. Free Amino Acid and Sugar Concentration

Most of the amino acid concentrations were significantly affected by the treatments (*p* < 0.05), except for aspartate, hydroxyproline, and cystine (*p* > 0.05, Table 1). No amino acids were found to be greater in the WA treatment compared to the DA treatment. The total free amino acids concentrations were significantly different among the samples (*p* < 0.05), with greater concentrations in all dry-aged samples compared to WA samples. When expressed using the dry-matter basis, DWA samples had the greatest concentration of total free amino acids (*p* < 0.05), followed by DA samples. In contrast, UDA and WA samples had the lowest total free amino acid concentrations (*p* < 0.05) which were not significantly different from each other (*p* > 0.05).

The sugar content was generally increased in the dry-aging treatment compared to the WA treatment (Table 2). Total sugar and reducing sugar content were increased in both DA and DWA steaks compared to WA steaks (*p* < 0.05), while UDA steaks were not different compared to all treatments (*p* > 0.05). Of the 10 sugars identified, ribose, glucose, and myoinositol were the only sugars identified to be significantly altered following the aging process. The ribose content was greatest in DA samples and lowest in WA samples (*p* < 0.05), while DWA and UDA samples had intermediate ribose concentrations and were not different from both DA and WA samples (*p* > 0.05). Similarly, myoinositol content was most abundant in DA steaks and lowest in WA steaks (*p* < 0.05). The glucose concentration was significantly higher in all dry-aged samples compared to the WA samples (*p* < 0.05). An analysis of the total sugars on a dry matter basis demonstrated a strong trend (*p* = 0.0535) of increasing sugar concentration in both DA and DWA loins compared to UDA and WA loins.

### 2.2. Free Fatty Acid and Volatile Content Analysis

Following free fatty acid profiling, 34 free fatty acids were identified and quantified, ranging from C10 to C24 (Table S1). A total of 7 free fatty acids (C13:1, C15:0, C17:1, C18:2trans, C18:3n3, C20:2, and C20:5) were affected by the aging treatments applied (*p* < 0.05). Of those significantly altered, most were identified as polyunsaturated fatty acids (C18:2trans, C18:3n3, C20:2, and C20:5), and were greater in DWA and/or UDA samples (*p* < 0.05). The overall percentage of saturated fatty acids (SFA), monounsaturated fatty acids (MUFA), and polyunsaturated fatty acids (PUFA), however, was not affected by the different aging treatments (*p* > 0.05). The total free fatty acids content was not affected when expressed on either a wet- or dry-matter basis (*p* > 0.05). However, a trend (*p* = 0.0689) for lower free fatty acid concentration was observed in UDA samples when expressed on a dry matter basis.

A total of 52 volatile compounds were detected, including 11 aldehydes, 6 alcohols, 6 ketones, 9 hydrocarbons, 4 pyrazines, 1 furan, 1 lactone, 6 sulfur-containing compounds, and 8 carboxylic acids (Table 3). Of those, the concentrations of 31 compounds were significantly affected by the different aging treatments (*p* < 0.05). The DWA samples consistently had the highest volatile compound concentrations among the different aging treatments, although the significance varied depending on the compounds. Among the dry-aging treatments, DWA and UDA were found to have significantly higher concentrations when compared to the DA treatment. The principal component analysis (PCA) did not reveal a distinct clustering between the aging treatments; however, the volatiles were found to be expressed and more correlated with the dry-aging treatments, particularly the DWA samples (Figure S1). Additionally, the aging treatments affected the hydrocarbon, alcohol, aldehyde, and ketone groups, as the majority of the significantly impacted volatile compounds originated from those groups.

### 2.3. Metabolomics Analysis

**Figure 1:**
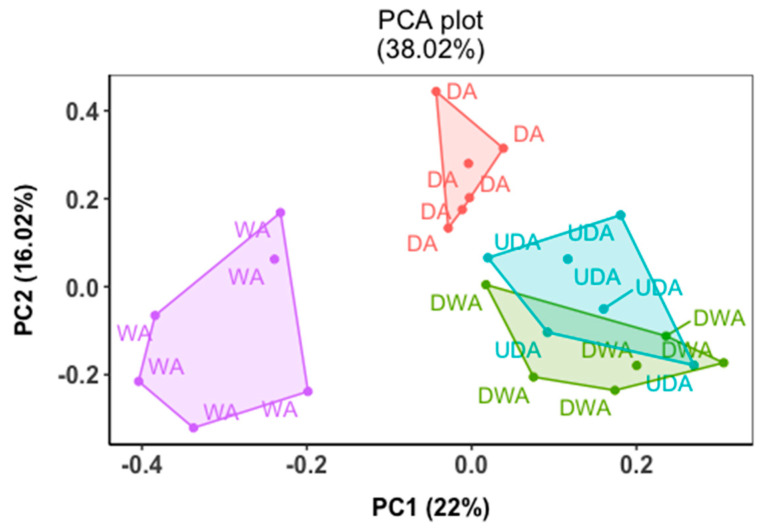
Principle component analysis (PCA) of significant metabolites from cull cow beef loins (M. longissimus lumborum) aged with different aging methods (wet aging (WA), dry-aging (DA), dry-aging in a water-permeable bag (DWA), and UV-light dry-aging (UDA)).

The metabolomics profiling was conducted via the ultra-performance liquid chromatography–mass spectrometry (UPLC-MS) platform. The analysis detected 1405 metabolite features across all the treatments. Of those metabolites, 60 metabolites were found to be significantly affected by the aging treatment applied (*p* < 0.05, FDR < 0.05) and were utilized for further analysis. The PCA of the metabolites exhibited a separation of the metabolite profile based on their treatments (Figure 1). A notable separation between all the dry-aging treatments and wet-aging treatments could be observed across the PC1 axis, explaining 22% of the variation observed. Additionally, the dry-aging treatments were further separated along the PC2 axis, with 16.02% of the variation being explained. The PC2 showed that DA samples were isolated from both DWA and UDA samples, indicating a distinct metabolites profile in the DA treatment. The separation across PC1 could be attributed to an increased abundance of Proline, Ile-Ile, Leu-leu-leu, Lysophosphatidylethanolamine, and Tetrahydrofurfuryl cinnamate in WA, separating the treatment from the dry-aging treatments (Table S2, Figure S2). For PC2, the clustering could potentially be attributed to the increased abundance of Gluthathionyl acetate, Asp-Cys, Thioproline, and Phenylethyl glucopyranoside in the DA treatments, explaining the separation observed between the different dry-aging treatments (Table S2, Figure S2)

Of the 60 significant features, 42 were able to be annotated through a mass comparison with the HMDB database and were then loosely categorized into protein-derived, carbohydrate-derived, lipid-derived, organic acids, and others (Table 4). The majority of the features identified belonged to the protein-derived group as amino acids and dipeptides. Most of these features were also presented in greater abundance in the dry-aging treatments than WA counterparts. Among the dry-aging treatments, the DA sample had more amino acids/dipeptides metabolites present in higher concentrations when compared to the DWA and UDA samples. Similarly, more carbohydrate and organic acid species were present in higher abundance in one of the dry-aging treatments compared to the WA treatment.

### 2.4. Microbiome Analysis

#### 2.4.1. Sequence Quality and Contamination

Following the 16S rRNA gene sequencing and quality control via DADA2, a total of 6,270,992 sequences were identified. The sequences were then able to be clustered into a total of 565 Amplicon Sequence Variants (ASVs) in the study. A comparison to PCR negative control samples (PCR-grade water used as DNA template) indicated the potential contamination of the bacterial genus identified as *Escherichia-Shigella*. The relative abundance of the genus was observed to be more than 95% in the negative control samples and therefore was considered a contaminant. As such, all members of the genus were removed from the samples. All samples were then rarified to a sampling depth of 2391 to minimize the removal of low sequence read samples and used for subsequent microbiome analyses. The initial samples were excluded from the analysis as most of the samples had very low sequences following the contaminant removal.

#### 2.4.2. Diversity Measures

In the current study, the alpha diversity was estimated using multiple measures, including the Chao1 index for richness, the Pielou index for evenness, and the Faith phylogenetic diversity index for phylogenetic diversity estimation. A significant treatment effect was observed for both Chao1 and Pielou index measures (*p* < 0.05, Figure S3), and a significant source effect was observed for the Faith phylogenetic diversity measure (*p* < 0.05, Figure S3). No significant interaction between treatment and source was observed across all alpha diversity measures (*p* > 0.05). WA samples had a greater richness (Chao1) when compared to the DWA samples (*p* < 0.05), while the DA and UDA samples were similar to both WA and DA samples (*p* > 0.05). Similarly, WA samples also had significantly higher evenness than DA samples (*p* < 0.05) but not when compared to DWA and UDA samples. The phylogenetic diversity was only influenced by the sample source with greater diversity in the crust samples when compared to lean samples (*p* < 0.05).

The Bray–Curtis Dissimilarity index and Weighted UniFrac were calculated to estimate beta diversity in this study. Similar results could be observed from the principal coordinate analysis (PCoA) of both measures, showing a clear separation between the dry-aging treatments and the wet-aging treatment community (Figure S4). Homogeneity analysis did not identify significant differences across all effects (*p* > 0.05), indicating similar sample dispersion within each of the treatments. Therefore, any community dissimilarity observed could be attributed to the separation of treatment group centroids. PERMANOVA of the community structure based on the Bray–Curtis index revealed a significant aging treatment and source interaction (*p* < 0.05). When phylogenetic relations were taken into consideration in the Weighted Unifrac index, a significant aging treatment effect (*p* < 0.05) and source effect (*p* < 0.05) were observed. Pairwise analysis of the Bray–Curtis index revealed significant community differences between both lean and crust samples of all dry-aged treatments when compared to both lean and crust portions of WA treatment (*p* < 0.05). Pairwise analysis of the Weighted Unifrac index demonstrated that the DA and DWA communities were similar (*p* > 0.05) and were both different from the WA community (*p* < 0.05). The DWA treatment was also found to have a significantly different bacterial community when compared to the UDA treatment (*p* < 0.05).

#### 2.4.3. Relative Abundances, Microbial Markers, and Co-Occurrence

**Figure 2:**
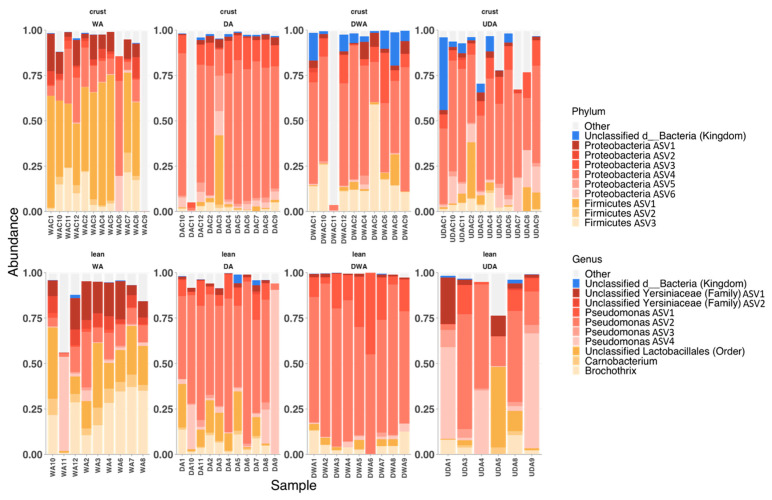
Relative abundances of the top 10 bacterial ASVs from cull cow beef loins (*M. longissimus lumborum*) aged with different aging methods (wet aging (WA), dry-aging (DA), dry-aging in a water-permeable bag (DWA), and UV-light dry-aging (UDA)). Amplicon sequence variant (ASV) identifications at both phylum and genus levels are indicated. For example, Pseudomonas ASV1 is classified as Proteobacteria at the phylum level.

The 10 ASVs with the highest relative abundances comprised about 90% of the microbial community of most samples (Figure 2). No pathogens were identified in the current study through the taxonomy identification. Similar ASV compositions were observed between the crust and lean portions of the same treatment, with greater consistency between replicates observed within the lean portion. The WA microbial community composition in both crust and lean portions was dominated by unclassified Lactobacillales, *Brochothrix*, and unclassified Yersiniaceae, comprising more than 50% of the total microbial abundances. Conversely, the crust and lean of the dry-aged samples were mainly comprised of *Pseudomonas* spp., with the genera presenting more than 50% of the total bacterial abundances.

The microbial marker analysis using LEFSE and ANCOM (Table S4) identified common microbes, which could potentially indicate their influence during the aging process. The *Pseudomonas* ASV1 was shown to be greatly enriched in the DA treatment, while unclassified Yersiniaceae ASV1, *Carnobacterium*, unclassified Lactobacillales, and *Brochothrix* were enriched in the WA treatment. Co-occurrence analysis between the microbial ASVs and the significant metabolites also showed greater numbers of unique ASVs–metabolite pairs in the DA treatment than in other treatments (Table S3). Among the correlated metabolites, the majority of the compounds were identified to belong to the protein-derived group.

## 3. Discussion

### 3.1. Flavor Precursors and Flavor Generation

Among the different flavor precursors, the availability of free amino acids has been suggested as an integral aspect of meat flavor generation, mainly due to their involvement in the Maillard reaction to generate flavor volatiles [9]. In the current study, greater concentrations of free amino acids were identified in the dry-aging treatments compared to WA treatment through free amino acid analysis. Multiple studies have constantly reported similar results in meats from different animal species [2,10,11], where free amino acids increase as a result of dry-aging treatment and such an increase is likely essential for the final dry-aged flavor generation. Further, similar to the current result, those studies also reported that a greater increase in umami-related amino acids (i.e., glutamate and glutamine) was observed, indicating the importance of such amino acids in developing the unique dry-aging flavor.

The contribution of the different amino acid groups to dry-aged flavor, however, is still unclear. A higher abundance of both cysteine and methionine in the dry-aged treatment was observed in the current study. Those amino acids were previously reported to generate a meat-like aroma volatile and positively correlated with beefy/meaty flavor in meat [12]. These results, however, were contradictory to the trained and consumer sensory analysis reported in our parallel study, which reported no difference in beefy flavor observed by the trained panel, and a more beefy flavor was observed in the WA samples by consumers [8]. In addition to generating flavor volatiles, free amino acids have been proposed to be a taste-active compound and, therefore, could also alter the aromas and taste perceived by the consumers [13].

The untargeted metabolomics analysis also revealed several dipeptides and short peptides in greater abundance in the DA treatments. The role of peptides in meat flavor generation, especially in the dry-aged product, is still not well studied. A previous study suggested that a peptide-based Maillard reaction will generate more volatile compounds compared to an amino acid-based Maillard reaction [14]. Likewise, peptides could also be a taste-active compound, influenced by their amino acid compositions. Several short peptides were also abundant in the WA treatments, mainly containing isoleucine, leucine, proline, hydroxyproline, and phenylalanine. The majority of these amino acids, with the exception of isoleucine and hydroxyproline, were identified to produce a bitter taste in peptides [15].

The concentration of reducing sugars available in meat will also play a major role in the flavor development as they participate in the Maillard reaction. Similar to the current results, several studies also reported a greater abundance of reducing sugar after the dry-aging process [10,11,16]. More ribose, fructose, mannose, glucose, and myoinositol were observed in beef loins dry-aged for 21 days compared to those wet-aged for 28 days [16]. In the current study, only ribose, glucose, and myoinositol were significantly affected by the aging treatments, although a general trend of increased sugar concentration in all dry-aging treatments was observed compared to the WA treatment equivalent. Among the sugars, ribose was often considered the primary sugar source involved in the Maillard reaction in meat products since this type of sugar could be released through the degradation of nucleotides such as inosine and adenosine [17,18]. However, in the present study, glucose was available in significantly higher concentrations when compared to ribose and other sugars (~0.66 mmol/Kg of wet meat compared to ~15.81 mmol/Kg of wet meat for average ribose and glucose concentrations, respectively). The current observation could indicate that glucose still plays a significant role in the Maillard reaction in dry-aged products, likely due to its relatively high abundance. It was suggested by Dinh et al. [19] that ribose sugar was more unstable and rapidly degraded when compared to glucose, thus potentially further explaining the lower ribose concentration observed in the current study. The elevated concentration of free amino acids and reducing sugars observed in the dry-aging treatments could promote more Maillard reactions during the cooking process. Supporting this speculation, more Maillard reaction-based volatile compounds such as Strecker aldehydes (2-methylbutanal and 3-methylbutanal), pyrazine (methyl-pyrazine), and sulfur-containing volatile compounds (carbon disulfide, dimethyl sulfide, and methanethiol) were identified and present in greater abundance in the dry-aging treatments, especially in the DWA samples.

The extent of flavor volatile production is often dependent on the free fatty acid profile of the meat products. With regards to the free fatty acid profile, limited information is available with respect to dry-aging in the current literature. Available studies reported no changes or minimal alteration in the free fatty acid profile following the dry-aging application [6,16,20]. In the present study, no differences were found in the total concentration of the free fatty acids among the different treatments (*p* > 0.05). However, differences were identified when observing the proportion of the free fatty acids between the aging treatments, indicating the potential alteration of free fatty acid composition through the application of dry-aging. A higher proportion of PUFAs was observed in DWA and UDA treatments when compared to both WA and DA treatments. Likewise, greater concentrations of volatile compounds were released from DWA and UDA samples after the cooking process. More lipid-based volatile compounds, such as hydrocarbons, alcohols, n-aldehydes, and ketones, were present in higher abundance in both DWA and UDA samples when compared to both WA and DA samples. The greater lipid volatile compounds production could be attributed to the greater proportion of unsaturated fatty acids in those treatments. Unsaturated fatty acids are more readily oxidized and degraded and thus more active during the lipid thermal oxidation and degradation during cooking [21].

Although greater concentrations of lipid volatile compounds were observed in the current study, lipid volatile compounds tend to have a higher detection threshold for influencing the final meat flavor [22]. Interestingly, the interaction between the Maillard reaction and lipid thermal oxidation/degradation may further enhance the abundance and variation of flavor volatiles generated during the cooking process [23]. It has been proposed that the lipid thermal oxidation/degradation products (e.g., aldehydes, acids) could participate in the Maillard reaction by acting as a substrate to generate unique meat flavor volatiles such as pyrazines and thiazoles [23,24]. Thus, it would be reasonable to postulate that the higher production of lipid volatile compounds along with greater Maillard reaction ability could affect the flavor potential of the dry-aged product. The increased interaction of both flavor production mechanisms might be translated to the greater concentration and variation of volatile compounds produced in the dry-aged product compared to the WA treatments, thus explaining the more desirable flavor often perceived from the dry-aged product.

### 3.2. Flavor Precursors Generation Mechanism

#### 3.2.1. Dehydration

Dehydration has been considered a major mechanism responsible for the flavor development in dry-aged meat products, mainly from the moisture loss during the aging process, which subsequently concentrated the flavor precursors in the product. The extensive moisture loss during dry-aging is inevitable and has been shown to reach up to 35% loss depending on the length of the dry-aging [1]. In the current study, the amino acid abundance and reducing sugar concentration were influenced by the dry-aging treatments, where concentrations of both precursor groups increased following the dry-aging application. In our parallel study [8], both DA and UDA treatments had the highest aging shrinkage, showing 12.09% and 12.44% moisture loss from the aging process, respectively. The DWA loins had an intermediate moisture loss, averaging 7.59%. Likewise, lower moisture content in trimmed lean portions was reported for both DA and UDA compared to WA and DWA. This decrease in moisture content could be partially responsible for the observed increase in free amino acids and the reduced sugar concentration found in the current study, subsequently affecting the dry-aged flavor development. The dehydration process likely increased the relative abundance of the precursors, thus potentially promoting more Maillard reactions during cooking. This observation, therefore, confirms the postulation regarding the significance of dehydration in the flavor generation process of dry-aged meat. Different from the free amino acids and reducing sugars, the total free fatty acid concentration was not impacted by the dry-aging application when compared to WA. Further study to elucidate the impact of dry-aging on fatty acids profile alteration would be of interest to understand the dry-aging impact on the liberation of lipid-based flavor precursors.

#### 3.2.2. Microbial Involvement

The involvement of microorganisms (bacteria, yeast, and mold) has been suggested to participate in the liberation of flavor precursors. Previous reports suggested the involvement of microorganisms in the liberation of flavor precursors via the release of exogenous proteolytic and lipolytic enzymes [7,25,26]. The current microbiome analysis demonstrated that the phylum Proteobacteria has the highest relative abundance in all dry-aging treatments. This phylum has been previously reported as the most prominent microbial phyla in dry-aged meat by Ribeiro et al. and Capouya et al. [25,26], more specifically the Pseudomonas genus. Likewise, a greater abundance of the Firmicutes phylum was also reported to be the dominant bacteria group in wet-aged samples by Ribeiro et al. [25], similar to the current observation. The Firmicutes present in meat are often identified as *Lactobacillus* spp., which are anaerobic bacteria often observed in vacuum-packaged meat [27]. While the relative abundances depended on the environmental conditions, the aforementioned microbe groups have been identified as spoilage bacteria in meat products by degrading available nutrients such as proteins and lipids to other products, including free amino acids, fatty acids, organic acids, esters, and aldehydes [28]. However, it is reasonable to postulate that the presence of members of the Proteobacteria phylum is more influential in the liberation of flavor precursors such as free amino acids and reducing sugars, as those compounds were enhanced in the dry-aging treatments, due to the greater abundance of this phylum.

As previously discussed, the dry-aging treatments had distinct flavor precursor compositions compared to their wet-aging counterpart. The metabolomics profiling exhibited an apparent clustering between the dry-aged and wet-aged samples, demonstrating differences between the aging methods. While this separation could be attributed to the environmental factors during the aging process, the dehydration process might not necessarily increase the flavor precursors’ total availability in dry-aged meat. When presented on a dry matter basis, a greater abundance of free amino acids and reducing sugars were observed in both DA and DWA samples, indicating the involvement of other mechanisms in liberating those flavor precursors. The UDA samples were found to have a concentration similar to WA samples when the flavor precursors were presented on a dry matter basis. This observation may indicate that the elevated liberation of flavor precursors could be attributed to the participation of microorganisms during the dry-aging process, as the UV light substantially decreased and suppressed the presence of microbes, as reported in our parallel study [8]. While UDA samples had a similar microbiome profile to both DA and DWA samples, the microbiome analysis used recovered DNA materials and did not distinguish between active, dead, or injured bacteria [29]. Therefore, while a similarity was observed, it is possible that the UV light suppressed the microbial activity, which could result in no considerable impact on the flavor precursor changes.

More protein-derived metabolites were identified to be correlated to more unique ASVs, indicating that the microbes might play a significant role in the protein degradation, such as the glutamyl peptides. These peptides were previously identified to be released through the activity of *Bacillus* spp. [15], which were also identified as a unique ASV–metabolites pair in the DA treatment. This could indicate that the activity of less abundant microbes (such as *Bacillus* spp.) present in the meat during dry-aging could also contribute to the overall flavor precursor development in the meat. Perhaps the major microorganism groups would increase the flavor precursors without any distinction, while the minor microorganism groups could be more specific and liberate more particular flavor precursors. This could lead to the generation of unique compounds that could potentially influence the final perceived flavor, such as the release of glutamyl peptides by the *Bacillus* spp. as observed in the current study. Future study on specific bacteria metabolism would be of interest to provide insight into the liberation of unique metabolites.

The role of mold and yeast was not analyzed in this study. However, a previous study has identified the involvement of mold (*Pilaira anomala*) and yeast (*Debaryomyces hansenii*) in liberating the flavor precursors [7]. Furthermore, our parallel study observed greater mold and yeast content in the DWA samples [8], indicating their potential activity in explaining the difference in free amino acids and free fatty acid profiles observed in the DWA samples compared to DA. Future studies to further identify this relation will be of interest to fully understand the impact of microorganisms in dry-aging flavor development.

## 4. Materials and Methods

### 4.1. Sample Collection, Preparation, and Processing

The sample collection process was described in our parallel study [8]. In brief, paired bone-in beef loins were collected from 13 carcasses (42+ months old, C maturity, Holstein, NAMP:175, M. *longissimus lumborum*) at 5 days postmortem. Prior to any processing, initial (INI) samples were individually excised from the loin eye area of one side of the loins for microbiome profiling. The loins were then split into 4 equal sections (average weight = 4500 g per section) and randomly assigned into 4 different aging treatments: wet-aging (WA; Clarity Vacuum Pouches, Bunzl Processor Division, Riverside, MO, USA.), conventional dry-aging (DA), dry-aging in a water-permeable bag (DWA; UMAi Dry^®^ Short Loin (Large), UMAI Dry, Minneapolis, MN, USA) and UV-light dry-aging (UDA). All samples were aged in a walk-in cooler for 28 days at 2 °C and 65% relative humidity with a 0.8 m/s airflow. The UDA samples were treated with UV light twice per day (Phillip TUV T8 UVC light, Eindhoven, The Netherlands). The UV lights were mounted 30 cm above the samples and turned on for a total of 5 min per treatment (totaling a dose of 5 J/m^2^ for each UV treatment). At the end of aging, samples were trimmed of the dehydrated surfaces (crust) and deboned. Following the trimming process, the crust/surface samples were collected and lean portion samples were excised from the section for microbiome profiling. The sections were then cut into steaks (2.4 cm thick) and collected for further biochemical analysis. All samples were individually vacuum packaged and stored in a −80 °C freezer until further analyses.

### 4.2. Free Amino Acid Analysis

The free amino acid analysis was performed using the method described by Vierck et al. [30]. The samples were first prepared and extracted following the technique used by Koutsidis et al. [18]. In brief, 3 g of homogenized sample was added to a conical tube containing 10 mL of cold water (deionized and autoclaved) and 300 µL of rhamnose (2 mg/mL) and was shaken for 10 min. The sample was then centrifuged, and the supernatant was collected. The pellet was then resuspended in 5 mL of cold water and recentrifuged following the same procedure. The supernatant from both extractions was combined and filtered through a 0.2 µm disc filter to remove any fat and/or tissue particles. The filtered sample was then derivatized using the EZ-Faast amino acids kit (Phenomenex, Torrance, CA, USA), following the manufacturer’s guidelines.

The free amino acid content was measured using gas chromatography–mass spectrometry (GC-MS) (Agilent Technologies, Palo Alto, CA, USA) in electron impact mode with a 3:1 split ratio. The derivatized sample was separated using a Zebron EZ-AAA Amino Acid GC Column (10 m × 0.25 mm × 0.15 mm; Phenomenex, Torrance, CA, USA) with helium as the carrier gas. Both internal standards (norvaline) and authentic standards for each amino acid were utilized to identify and quantify the free amino acids from the samples. Concentrations were then reported as millimoles per kilogram of the initial wet sample.

### 4.3. Sugar Content Analysis

Prior to the sugar analysis, meat samples were extracted following the method by Koutsidis et al. [18] as described in the previous section. After the extraction process, the liquid extract was freeze-dried, and the final dried product was added to a solution containing dimethyl sulfoxide, hexamethyldisilazane, trimethylchlorosilane, and cyclohexane. The sample was sonicated and incubated at room temperature for 24 h. Following incubation, the organic layer was separated and injected into GC-MS (Agilent Technologies, Palo Alto, CA, USA) in electron impact mode. The gas chromatograph was set to splitless mode, and the injector temperature was set to 250 °C. The oven temperature was initially set at 60 °C for 1 min, increased to 130 °C for 2 min, followed by a 2 °C/minute increase until 170 °C, and finally adjusted to 300 °C by gradually increasing the temperature by 4 °C/minute. The separation was performed using the DB-17 ms capillary column (30 m × 0.25 mm; 0.25-μm film thickness) coupled with 1.5 mL of deactivated methylsilicone-fused silica capillary retention gap. Helium was utilized as the carrier gas. Authentic standards (Sigma-Aldrich, Bellefonte, PA, USA) were used to identify peaks, and rhamnose was utilized as an internal standard to quantify the sugar concentration. The sugar concentration was reported in millimoles per kilogram of the initial wet sample.

### 4.4. Free Fatty Acid Analysis

The free fatty acid content was analyzed using the protocol described by Chail et al. [31]. The free fatty acids methyl esters (FAME) were prepared by incubating 1 g of the homogenized sample at 55 °C with internal samples (tridecanoic acid; 0.5 mg/mL in methanol) following the method described by O’Fallon et al. [32]. Hexane was then added to the vial and the sample was centrifuged to extract the FAME.

The extract (1 µL) was injected into GC equipment for analysis. The inlet was maintained at 250 °C with a 50:1 split ratio. Separation was performed using an HP-88 capillary column (100 m × 250 µm × 0.2 µm), and helium was used as a carrier gas with a flow rate set to 2.5 mL per minute. All free fatty acids were identified by comparing their retention time to GC internal reference standards (Nu-Chek Prep, Inc, Elysian, MN, USA). The free fatty acid concentration was presented as the percent of total free fatty acids.

### 4.5. Volatile Compound Analysis

The volatile compounds were profiled using the method outlined by Gardner and Legako [33]. Briefly, samples were cooked until the internal temperature reached 63 °C using a dual-sided clamshell grill (Griddler GR-150, Cuisinart, Glendale, AZ, USA), and six cores (1.27 cm in diameter) perpendicular to the muscle fibers were collected. The cores were minced using a coffee grinder (Mr. Coffee, Sunbeam Corporation, Boca Raton, FL, USA), and 5 g of the minced sample were transferred into vials. An internal standard (1,2-dichlorobenzene) was added to each vial and incubated for 5 min at 65 °C in a gerstel automatic sampler (Gerstel Inc., Linthicum Heights, MD, USA) followed by 20 min of extraction via headspace solid-phase microextraction. The volatile compounds extracted from the headspace were injected into a VF-5 MS capillary column (30 m × 0.25 mm × 1.0 µm; Agilent Technologies, Inc., Santa Clara, CA, USA) for separation and identification. Identified volatiles were compared to authentic standards (Sigma-Aldrich, St. Louis, MO, USA) for validation. The volatile compound concentrations were reported in nanograms per gram of the initial wet sample.

### 4.6. Metabolomics Profiling

#### 4.6.1. Sample Extraction

A total of 6 samples were randomly selected from each of the aging treatments for the metabolomics analysis. The samples were homogenized by submerging the samples into liquid nitrogen and powdered using a blender (Waring Products, CT, USA). The metabolites were then extracted using the method described by Setyabrata et al. [6]. In brief, 100 mg of each sample was extracted using an equal amount of chloroform (300 µL) and methanol (300 µL) in a Precellys 24 tissue homogenizer (Bertin Instruments, Bretonneux, France). The homogenizer extraction was conducted in 3 cycles of 30 s at 6500 rpm with 30 s rest. After the homogenization process, water was added, and the mixture was centrifuged at 16,000× *g* for 8 min. The upper layer was collected and dried for chromatographic separation.

#### 4.6.2. Ultra-Performance Liquid Chromatography–Mass Spectrometry Analysis (UPLC_MS)

A non-targeted metabolomics analysis was conducted according to the procedure described by Setyabrata et al. [6]. The dried samples were first reconstituted into an aqueous solution containing 95% water, 5% acetonitrile, and 0.1% formic acid. The reconstituted samples were then assayed using an Agilent 1290 Infinity II UPLC system (Agilent Technologies, Palo Alto, CA, USA) equipped with a Waters Acquity HSS T3 (2.1 × 100 mm × 1.8 µm) separation column (Waters, Milford, MA, USA) for separation. The column was maintained at 40 °C with the binary mobile phase flow set at 0.45 mL/minute. The binary mobile phase consisted of solvent A (0.1% formic acid (*v*/*v*) in ddH2O) and solvent B (0.1% formic acid (*v*/*v*) in acetonitrile). Initial conditions of 100:0 A:B were held for 1 min, followed by a linear gradient to 70:30 over 15 min, changed to a linear gradient of 5:95 over 5 min, and 5:95 held for 1.5 min.

Following the separation, the sample was identified using Agilent 6545 quadrupole time-of-flight (Q-TOF) mass spectrometer (Agilent Technologies, Santa Clara, CA, USA) with positive electrospray ionization (ESI) mode for data collection. HPLC-MS scans were collected over a range of 70–1000 m/z. HPLC-MS-MS data was collected to aid in compound identification. The collected data were analyzed using Agilent MassHunter B.06 software (Agilent Technologies, Santa Clara, CA, USA), and the mass accuracy was improved by infusing Agilent Reference Mass Correction Solution (G1969-85001; Agilent Technologies, Santa Clara, CA, USA). The peak deconvolution was conducted using Agilent ProFinder (Agilent Technologies, Santa Clara, CA, USA) and annotated using the HMDB (www.hmdb.ca (accessed on 15 September 2021)) metabolite database.

### 4.7. Microbiome Analysis

#### 4.7.1. Sample Preparation and DNA Extraction

Meat samples (5 g) were aseptically collected, in a stomacher bag (WhirlPak, Madison, WI, USA) containing 50 mL of sterile 0.1% peptone water and stomached by hand for 1 min. The rinsate was then collected and centrifuged at 3200× *g* for 40 min. After the centrifugation, the supernatant was removed, and the pellet was resuspended in 1.5 mL of sterile 0.1% peptone water before centrifuging the samples at 21,000× *g* for 10 min. Following the second centrifugation, the supernatant was removed and the pellet was stored at −80 °C until further processing. Total DNA extraction was conducted using the DNeasy PowerLyzer PowerSoil Kit (Qiagen, Germantown, MD, USA), following the manufacturer’s guidelines.

#### 4.7.2. 16S Library Preparation and Sequencing

The library was constructed by PCR using the barcode indexed amplification product from the V4 region of the 16S rRNA using AccuPrime™ Pfx SuperMix (Thermo Scientific, Waltham, MA, USA) as described by Kozich et al. [34]. The PCR amplicon quality was then checked via gel electrophoresis. The amplified DNA was then normalized using the SequalPrep™ Normalization Plate Kit (Thermo Scientific, Waltham, MA, USA) following the manufacturer’s guidelines. Finally, samples were pooled by collecting 5 μL of the amplified DNA from each sample for amplicon sequencing via the Illumina MiSeq sequencing platform (2 × 250 paired-end; Illumina Inc., San Diego, CA, USA). All sequences were deposited and accessible in the NCBI sequence read archive database under Bioproject PRJNA823742 (https://www.ncbi.nlm.nih.gov/bioproject/?term=PRJNA823742 (accessed on 17 April 2022)).

#### 4.7.3. Bioinformatics Analysis

The raw sequences obtained were analyzed using Quantitative Insight into Microbial Ecology (QIIME2) v.2020.2. The samples were denoised using the DADA2 step [35] with both the forward and reverse sequences trimmed at position 0 and truncated at position 245 to obtain sequences with qualities of >Q30. All the sequence reads were clustered into Amplicon Sequence Variants (ASVs) with 100% similarity to identify unique microbiome variants. The sequences were then rarefied with a sampling depth of 2391 for both alpha and beta diversity calculation. Both alpha and beta diversity metrics were estimated using the QIIME2 pipeline, measuring the Chao1 index (richness), the Pielou index (evenness), and the Faith phylogenetic diversity index (phylogenetic diversity estimation) for alpha diversity and the Bray–Curtis Dissimilarity index and Weighted UniFrac for the beta diversity. The taxonomy was assigned by matching to the SILVA 13_8, 515F/806 region database. Files utilized in the data analysis are available at https://github.com/dsetyabr/MeatMicrobiome (accessed on 17 April 2022).

### 4.8. Statistical Analysis

This study utilized a randomized complete block design with the different aging treatments as the fixed effect and animals as the random effect. Source location (crust/surface and lean) was added as an additional fixed effect for the microbiome analysis to consider the potential location effect. The free fatty acid, free amino acid, reducing sugar, and volatile compounds concentrations were analyzed using the PROC GLIMMIX procedure of the SAS 9.4 software (SAS Institute Inc., Cary, NC, USA). The least-square means for all traits were separated, and the statistical significance level was defined at the level of *p* < 0.05. An unsupervised principal component analysis was performed to further analyze the volatile compounds.

The metabolomics data were analyzed using RStudio (Boston, MA, USA). The metabolite peaks were normalized using log 2 transformation and were checked for the presence of extreme variance within the group. The metabolites were also analyzed using ANOVA to identify features significantly affected by the aging treatment. Significance was defined at *p* < 0.05 and adjusted using the false discovery rate (FDR) method. An unsupervised principal component analysis was performed to visualize the data.

The alpha and beta diversity of the microbiome data were visualized using RStudio (Boston, MA, USA) using metrics generated by QIIME2. The permutational multivariate analysis of variance (PERMANOVA; *p* ≤ 0.05) and multivariate homogeneity analysis to test the difference in beta diversity were performed using the vegan package [36]. The significance was set at *p* < 0.05 and adjusted using the false discovery rate (FDR) method. The co-occurrence analysis was also performed to identify the ASV–metabolites pairs most prevalent within each aging treatment. The significant metabolites and all identified ASVs were utilized in the co-occurrence analysis. Significant ASV–metabolites pairs were determined at *p* < 0.05 and R^2^ > 0.8. Linear discriminant analysis effect size (LEFSE) and Analysis of Compositions of Microbiomes (ANCOM) were also performed to identify potential microbial markers unique to the different aging methods. Differences were considered statistically significant at *p* < 0.05 and adjusted using the FDR method.

## 5. Conclusions

The results of the current study demonstrated that dry-aging increased the abundance of flavor precursors, such as free amino acids, short peptides, and reducing sugars, which are key chemical compounds related to Maillard reactions. While only a limited impact was observed in the free fatty acid profiles, more PUFAs were identified in DWA and UDA samples, potentially contributing to the greater lipid volatile compounds as a result of those treatments. Greater flavor volatiles were observed in the dry-aging samples, especially in DWA samples, likely due to greater chemical interactions between compounds related to the Maillard and lipid chemical reactions. Two major dry-aging flavor precursor generation mechanisms, dehydration and microbial-induced liberation, were characterized in the current study. While dehydration played a role in increasing overall flavor precursors concentration, it did not influence their relative abundance. Microbiome analysis revealed that microbial groups, especially Proteobacteria, might contribute to the increased availability of the flavor precursors in dry-aged treatments. The microbiome co-occurrence analysis also identified minor microbial groups which could potentially release unique metabolites that contribute to the overall dry-aged flavor. Future studies to identify the role of the mold and yeast will be of interest to identify their role in dry-aged flavor development.

## Figures and Tables

**Table 1:** Effect of different aging methods on the free amino acid contents of cull cow beef loins (*M. longissimus lumborum*) after 28 days of aging.

Free Amino Acids (mmol/Kg of Wet Meat)	WA	DA	DWA	UDA	SEM	*p*-Value
Alanine	3.899 ^b^	6.477 ^a^	5.730 ^a^	5.601 ^a^	0.345	<0.0001
Asparagine	0.218 ^c^	0.323 ^ab^	0.348 ^a^	0.269 ^bc^	0.027	0.0021
Aspartate	0.103	0.153	0.165	0.145	0.022	0.0622
Beta-Alanine	0.241 ^b^	0.349 ^a^	0.315 ^ab^	0.386 ^a^	0.040	0.0498
Cysteine	1.056 ^b^	1.578 ^a^	1.685 ^a^	1.398 ^a^	0.129	0.0008
Cystine	0.015	0.016	0.016	0.014	0.003	0.9738
Glutamate	0.920 ^c^	1.586 ^b^	2.278 ^a^	1.287 ^b^	0.139	<0.0001
Glutamine	0.002 ^c^	0.012 ^a^	0.011 ^a^	0.006 ^b^	0.001	<0.0001
Glycine	1.329 ^b^	2.153 ^a^	1.961 ^a^	1.859 ^a^	0.140	0.0004
Histidine	1.050 ^b^	2.773 ^a^	3.675 ^a^	3.164 ^a^	0.352	<0.0001
Hydroxyproline	0.041	0.047	0.048	0.057	0.005	0.1702
Isoleucine	0.846 ^b^	1.134 ^a^	1.130 ^a^	1.059 ^ab^	0.095	0.0415
Leucine	1.065 ^b^	1.396 ^a^	1.256 ^ab^	1.340 ^a^	0.103	0.0462
Lysine	0.477 ^c^	1.353 ^a^	1.308 ^a^	1.002 ^b^	0.115	<0.0001
Methionine	0.379 ^b^	0.606 ^a^	0.523 ^a^	0.520 ^a^	0.052	0.0048
Ornithine	0.048 ^b^	0.118 ^a^	0.110 ^a^	0.083 ^ab^	0.013	0.0017
Phenyl Alanine	0.508 ^b^	0.749 ^a^	0.721 ^a^	0.720 ^a^	0.062	0.0057
Proline	0.356 ^b^	0.483 ^a^	0.476 ^a^	0.415 ^ab^	0.033	0.0206
Serine	1.336 ^c^	2.070 ^b^	2.726 ^a^	2.029 ^b^	0.213	0.0001
Threonine	0.767 ^c^	1.159 ^ab^	1.394 ^a^	1.113 ^b^	0.103	0.0007
Tyrosine	0.389 ^b^	0.840 ^a^	0.825 ^a^	0.769 ^a^	0.074	<0.0001
Tyrptophan	0.047 ^b^	0.102 ^a^	0.087 ^a^	0.083 ^a^	0.009	0.0004
Valine	1.409 ^b^	1.875 ^a^	1.865 ^a^	1.729 ^ab^	0.148	0.0454
Total Free Amino Acid	16.308 ^b^	27.351 ^a^	28.652 ^a^	24.522 ^a^	1.806	<0.0001
Total Free Amino Acid Dry Basis (mmol/Kg dry meat)	50.663 ^c^	69.480 ^b^	85.513 ^a^	53.074 ^c^	5.226	<0.0001

^a–c^ Different superscript letters indicate a significant difference between the different aging methods (*p* < 0.05). Different aging treatments: wet-aging (WA), conventional dry-aging (DA), dry-aging in a water-permeable bag (DWA), and UV-light dry-aging (UDA). SEM: Standard Error of Means.

**Table 2:** Effect of different aging methods on the sugar concentration of cull cow beef loins (*M. longissimus lumborum*) after 28 days of aging.

Reducing Sugars (mmol/Kg of Wet Meat)	WA	DA	DWA	UDA	SEM	*p*-Value
Ribose	0.5173 ^b^	0.7881 ^a^	0.6672 ^ab^	0.6808 ^ab^	0.0690	0.0418
Fructose	0.8581	1.3355	1.1871	1.1818	0.1743	0.2260
Mannose	1.8872	2.7303	2.7838	2.5197	0.2789	0.0734
Glucose	11.1989 ^b^	19.4053 ^a^	16.1550 ^a^	16.4701 ^a^	1.4932	0.0017
Myoinositol	0.3815 ^c^	0.6911 ^a^	0.6224 ^ab^	0.4802 ^bc^	0.0703	0.0046
Ribose 5-phosphate	0.0467	0.0875	0.1674	0.0680	0.0343	0.0771
Fructose 6-phosphate	1.6241	2.0178	2.7800	1.7763	0.5367	0.4410
Mannose 6-phosphate	0.9645	1.4169	1.4313	1.4415	0.3009	0.5632
Glucose 6-phosphate	7.2096	13.1020	10.3266	8.7132	1.8726	0.1276
Maltose	0.0492	0.0603	0.0438	0.0468	0.0152	0.8184
Total Sugars	24.7371 ^b^	41.6349 ^a^	36.1647 ^a^	33.3785 ^ab^	3.8001	0.0222
Total Reducing Sugars	24.3556 ^b^	40.9438 ^a^	35.5423 ^a^	32.8983 ^ab^	3.7877	0.0251
Total Sugars Dry Basis (mmol/Kg of dry meat)	78.9235	105.82	108.33	73.6774	11.2584	0.0535

^a–c^ Different superscript letters indicate a significant difference between the different aging methods (*p* < 0.05). Different aging treatments: wet-aging (WA), conventional dry-aging (DA), dry-aging in a water-permeable bag (DWA), and UV-light dry-aging (UDA). SEM: Standard Error of Means.

**Table 3:** Effect of different aging methods on the volatile compound profiles of cull cow beef loins (*M. longissimus lumborum*) after 28 days of aging.

Volatile Compounds Name (ng/g Sample)	WA	DA	DWA	UDA	SEM	*p*-Value
***n-aldehydes***						
Acetaldehyde	9.12 ^c^	19.22 ^c^	53.58 ^a^	36.99 ^b^	5.18	<0.0001
Butanal	4.67 ^c^	22.02 ^b^	44.26 ^a^	44.04 ^a^	5.54	<0.0001
Heptanal	8.99	8.70	17.06	7.72	3.13	0.1397
Hexanal	119.12	79.55	175.56	88.48	48.16	0.4940
Nonanal	6.74 ^ab^	4.4 ^b^	10.96 ^a^	2.72 ^b^	1.93	0.0245
Octanal	1.69 ^b^	2.32 ^b^	4.48 ^a^	2.16 ^b^	0.44	0.0002
Pentanal	40.86	3.41	8.28	4.21	19.87	0.4931
***Strecker aldehydes***						
2-methylbutanal	8.00 ^c^	49.59 ^b^	110.07 ^a^	106.20 ^a^	16.23	<0.0001
3-methylbutanal	10.29 ^c^	67.42 ^b^	144.84 ^a^	143.90 ^a^	21.82	<0.0001
Benzaldehyde	27.55	19.14	23.35	21.99	5.26	0.7209
Phenylacetaldehyde	2.94	1.74	2.31	1.45	0.56	0.2062
***Alcohols***						
1-Hexanol	1.11 ^c^	3.54 ^b^	5.60 ^a^	3.78 ^ab^	0.76	0.0007
1-Octanol	2.90 ^b^	2.38 ^b^	6.12 ^a^	1.06 ^b^	0.99	0.0061
1-Octen-3-ol	2.68 ^b^	5.63 ^ab^	7.74 ^a^	5.70 ^ab^	1.28	0.0262
1-Pentanol	12.83	14.89	23.77	26.65	6.20	0.3014
1-penten-3-ol	0.10 ^b^	0.64 ^ab^	1.12 ^a^	0.98 ^a^	0.21	0.0043
Ethanol	59.84	89.84	277.57	136.94	59.87	0.0635
***Ketone***						
2,3-butanedione	8.17 ^b^	54.37 ^a^	70.71 ^a^	65.68 ^a^	15.03	0.0198
2,3-pentanedione	0.04 ^b^	0.14 ^a^	0.19 ^a^	0.14 ^a^	0.02	0.0002
2-heptanone	1.69	2.25	2.89	2.44	0.48	0.3274
2-pentanone	0.41 ^c^	1.28 ^bc^	2.79 ^a^	1.79 ^ab^	0.35	0.0002
2-Propanone	42.12 ^b^	63.01 ^b^	131.49 ^a^	114.52 ^a^	13.86	<0.0001
3-hydroxy-2-butanone	10.88 ^b^	60.72 ^ab^	169.82 ^a^	133.57 ^a^	39.62	0.0223
***Hydrocarbon***						
Alpha-pinene	0.00 ^c^	0.13 ^cb^	0.35 ^a^	0.25 ^ab^	0.05	<0.0001
Benzene	0.99 ^b^	2.07 ^a^	2.58 ^a^	2.65 ^a^	0.31	0.0011
D-limonene	10.67 ^b^	30.01 ^a^	37.29 ^a^	30.31 ^a^	3.81	<0.0001
Ethyl benzene	0.37 ^b^	0.86 ^a^	1.06 ^a^	0.79 ^a^	0.13	0.0025
*p*-Xylene	0.97 ^b^	2.07 ^ab^	2.96 ^a^	2.22 ^a^	0.41	0.0083
Styrene	1.24 ^b^	2.38 ^a^	2.90 ^a^	2.46 ^a^	0.31	0.0028
Toluene	6.59 ^b^	17.03 ^a^	21.87 ^a^	20.13 ^a^	2.15	<0.0001
Octane	2.13 ^c^	6.13 ^b^	10.71 ^a^	7.96 ^ab^	1.02	<0.0001
Pentane	3.85 ^b^	5.46 ^b^	13.48 ^a^	10.56 ^a^	1.63	0.0003
***Pyrazine***						
2,5-dimethylpyrazine	5.69	4.89	6.64	5.25	2.04	0.9229
2-ethyl-3,5/6-dimethylpyrazine	1.48	1.41	2.08	1.56	0.45	0.6246
Methyl-pyrazine	0.97 ^b^	2.77 ^a^	3.91 ^a^	3.26 ^a^	0.78	0.0107
Trimethylpyrazine	1.08	1.30	2.35	1.58	0.46	0.1949
***Furans***						
2-Pentyl furan	0.92	0.45	0.69	0.38	0.28	0.5207
***Lactone***						
Butyrolactone	2.09 ^b^	15.94 ^a^	24.46 ^a^	18.11 ^a^	3.54	0.0004
***Sulfur-containing***						
2-methyl thiophene	0.59	0.47	0.59	0.51	0.05	0.2327
Carbon disulfide	10.33 ^b^	12.38 ^b^	21.11 ^a^	13.36 ^b^	2.57	0.0232
Dimethyl sulfide	3.74 ^c^	7.37 ^cb^	14.82 ^a^	10.82 ^ab^	1.43	<0.0001
Dimethyl-disulfide	0.03	0.03	0.06	0.05	0.01	0.1275
Methanethiol	2.88 ^bc^	1.54 ^c^	5.22 ^a^	4.12 ^ab^	0.77	0.0107
Methional	3.17	2.36	3.58	2.50	1.20	0.8636
***Carboxylic acid***						
Acetic acid	9.30 ^c^	11.25 ^cb^	23.11 ^a^	18.27 ^ab^	2.52	0.0008
Butanoic acid	20.30 ^c^	110.38 ^b^	202.04 ^a^	164.57 ^ab^	26.03	0.0001
Butanoic acid, methyl ester	3.55	0.98	1.55	2.01	1.67	0.6963
Heptanoic acid, methyl ester	0.17	0.24	0.37	0.27	0.07	0.2516
Hexanoic acid, methyl ester	3.51	4.57	6.70	6.70	1.53	0.3502
Hexanoic acid, methyl ester	3.11	4.57	6.70	6.70	1.53	0.2768
Nonanoic acid, methyl ester	0.79	0.46	0.53	0.34	0.13	0.0861
Octanoic acid, methyl ester	1.32	1.48	1.84	1.09	0.20	0.0690

^a–c^ Different superscript letters indicate a significant difference between the different aging methods (*p* < 0.05). Different aging treatments: wet-aging (WA), conventional dry-aging (DA), dry-aging in a water-permeable bag (DWA), and UV-light dry-aging (UDA) SEM: Standard Error of Means.

**Table 4:** Effect of different aging methods on the metabolomics profile of cull cow beef loins (*M. longissimus lumborum*) after 28 days of aging. (*p*-value < 0.05, FDR < 0.05).

Mass	RT	Highest Abundant	HMDB ID	Putative Name	WA	DA	DWA	UDA
***Protein-derived***								
115.0633	1.21	WA	HMDB0000162	Proline	18.32 ^a^	16.23 ^b^	16.92 ^b^	16.74 ^b^
244.1774	6.35	WA	HMDB28910	Ile-Ile	18.37 ^a^	16.21 ^b^	16.25 ^b^	16.49 ^b^
357.2623	9.86	WA	HMDB0094648	Leu-Leu-Leu	18.02 ^a^	16.30 ^b^	16.26 ^b^	16.65 ^b^
244.1067	7.38	WA/DA	HMDB0028864	Hyp-Hyp	17.91 ^a^	17.83 ^a^	17.06 ^b^	17.25 ^ab^
312.1437	5.47	WA	HMDB0131468	Phe-Phe	18.07 ^a^	17.94 ^ab^	16.98 ^c^	17.23 ^bc^
239.0794	7.21	DA/WA	HMDB0131468	Aspartic Acid	18.11 ^a^	18.12 ^a^	17.21 ^b^	17.53 ^b^
284.1122	3.95	DA/WA	HMDB0028821	Gln-His	19.11 ^a^	19.16 ^a^	18.19 ^b^	18.27 ^b^
204.1112	2.23	DA	HMDB0029136	Val-Ser	18.11 ^b^	18.50 ^a^	17.55 ^c^	17.59 ^bc^
236.0465	4.78	DA	HMDB0028750	Asp-Cys	17.66 ^ab^	17.96 ^a^	17.07 ^b^	17.24 ^b^
133.0196	4.78	DA	HMDB0062164	Thioproline	17.25 ^ab^	17.49 ^a^	16.62 ^b^	16.81 ^b^
218.1259	4.01	DA/DWA/UDA	HMDB0029042	Ser-Ile	20.92 ^b^	21.69 ^a^	21.40 ^a^	21.51 ^a^
284.11	1.71	DA	HMDB0028884	His-Glu	22.40 ^ab^	22.73 ^a^	21.74 ^b^	21.72 ^b^
174.1032	1.43	DA	HMDB0028854	Theanine	17.97 ^b^	18.33 ^a^	17.84 ^b^	17.66 ^b^
188.1165	3.43	DA/DWA	HMDB0000446	Acetyl-Lysine	22.50 ^b^	23.08 ^a^	23.08 ^a^	22.56 ^ab^
127.0632	8.22	DA/WA	HMDB0029434	Methyleneproline	17.53 ^a^	17.76 ^a^	16.83 ^b^	17.25 ^ab^
115.0634	0.86	DWA/WA	HMDB0000162	Proline	19.35 ^a^	19.10 ^b^	19.36 ^a^	18.85 ^b^
257.1022	0.82	DWA/WA	HMDB0039229	Gln-Gln	17.44 ^a^	16.99 ^b^	17.67 ^a^	17.13 ^ab^
155.0693	3.26	UDA/DWA/DA	HMDB0000177	Histidine	17.42 ^b^	17.99 ^a^	18.10 ^a^	18.32 ^a^
343.1257	1.85	UDA/DWA/DA	HMDB0037845	Deoxyfructosyl Tyrosine	20.48 ^b^	20.36 ^a^	20.86 ^a^	20.96 ^a^
***Carbohydrates-derived***								
464.2283	12.43	WA	HMDB0031367	Linalooloxide apiosylglucoside	21.06 ^a^	20.74 ^ab^	19.64 ^c^	20.21 ^bc^
284.1211	0.72	DA	HMDB0029819	Phenylethyl glucopyranoside	17.82 ^b^	18.28 ^a^	18.15 ^b^	18.19 ^ab^
379.1063	3.34	UDA/DA	HMDB0001066	Lactoylglutathione	19.40 ^b^	19.86 ^a^	19.68 ^ab^	20.22 ^a^
***Lipids-derived***								
565.4201	16.36	WA	HMDB0011497	Lysophosphatidylethanolamine	20.43 ^a^	19.18 ^b^	19.25 ^b^	19.47 ^b^
452.3357	14.23	WA/DWA/UDA	HMDB0037065	Oxoursadienoate	20.29 ^a^	18.96 ^b^	19.09 ^a^	19.42 ^a^
452.3361	14.04	WA	HMDB0035888	Tyromycic acid	21.83 ^a^	20.82 ^b^	20.82 ^b^	21.13 ^ab^
284.1073	1.56	WA/DA	HMDB0030694	Demethoxymatteucinol	21.66 ^a^	21.56 ^a^	20.89 ^b^	20.88 ^b^
232.1129	2.2	WA	HMDB0036189	Tetrahydrofurfuryl cinnamate	19.17 ^a^	18.81 ^ab^	18.43 ^b^	18.69 ^b^
286.1532	16.3	DA	HMDB0060085	Estradiol quinone	19.62 ^ab^	19.63 ^a^	18.64 ^c^	19.00 ^bc^
266.1728	12.08	UDA/DWA/DA	HMDB0030356	Didehydrocondyfolan	16.30 ^b^	17.32 ^a^	17.79 ^a^	18.04 ^a^
407.0982	4.11	UDA	HMDB0030257	Erysothiopine	19.98 ^ab^	19.46 ^b^	20.00 ^ab^	20.33 ^a^
132.0946	1.59	UDA/DWA	HMDB0029641	Cymenene	22.03 ^b^	22.06 ^b^	22.28 ^a^	22.29 ^a^
***Organic acids***								
365.0897	4.89	DA	HMDB0062198	Glutathionyl acetate	21.88 ^b^	22.24 ^a^	21.23 ^b^	21.42 ^b^
298.1283	3.01	DA/WA	HMDB06101	Enterolactone	17.90 ^a^	17.95 ^a^	16.93 ^b^	17.06 ^ab^
276.1212	3.05	DWA/DA	HMDB0034263	Triethyl citrate	22.12 ^b^	22.46 ^a^	22.51 ^a^	22.27 ^ab^
118.0277	3.59	UDA/DA/WA	HMDB0031204	Hydroxyoxobutanoic acid	18.57 ^a^	18.63 ^a^	18.44 ^b^	18.65 ^a^
164.0469	2.34	UDA	HMDB0001713	Coumaric acid	21.85 ^b^	21.77 ^b^	21.32 ^c^	22.13 ^a^
***Other***								
113.0843	6.55	WA	HMDB0031199	Trimethyloxazoline	21.64 ^a^	20.32 ^b^	21.13 ^ab^	20.32 ^b^
301.1637	4.59	WA/DA	HMDB0032654	Futoamide	19.15 ^a^	19.10 ^a^	18.43 ^b^	18.59 ^ab^
194.1156	5.62	DA/DWA/UDA	HMDB0094708	Tetraethylene glycol	22.17 ^b^	22.74 ^a^	22.56 ^a^	22.69 ^a^
132.0949	1.6	DWA	HMDB0032303	Heptanethiol	22.96 ^b^	23.12 ^a^	23.19 ^b^	23.11 ^b^
94.0395	1.53	DWA	HMDB0000228	Phenol	18.66 ^b^	18.76 ^b^	18.96 ^a^	18.71 ^b^
327.1884	4.55	UDA	HMDB0038645	Piperamide	20.47 ^ab^	19.89 ^b^	20.74 ^ab^	21.03 ^a^

^a–c^ Different superscript letters indicate a significant difference between the different aging methods (*p* < 0.05). Different aging treatments: wet-aging (WA), conventional dry-aging (DA), dry-aging in a water-permeable bag (DWA), and UV-light dry-aging (UDA).

## Data Availability

The microbiome sequence data and scripts utilized in the data analysis are available and can be accessed at https://www.ncbi.nlm.nih.gov/bioproject/?term=PRJNA823742 and https://github.com/dsetyabr/MeatMicrobiome (accessed on 17 April 2022).

## Supplementary Materials

The following supporting information can be downloaded at: https://www.mdpi.com/article/10.3390/metabo12060472/s1, Figure S1: Principal component analysis (PCA) biplot of volatile compounds from cull cow beef loins (M. longissimus lumborum) aged with different aging methods [Wet aging (WA), Dry aging (DA), Dry aging in water-permeable bag (DWA) and UV-light dry-aging (UDA)]; Figure S2: Principal component analysis (PCA) biplot of metabolites from cull cow beef loins (M. longissimus lumborum) aged with different aging methods [Wet aging (WA), Dry aging (DA), Dry aging in water-permeable bag (DWA) and UV-light dry-aging (UDA)]; Figure S3: Microbiome alpha diversity index on microbial community collected from cull cow beef loins (M. longissimus lumborum) aged with different aging methods [Wet aging (WA), Dry aging (DA), Dry aging in water-permeable bag (DWA) and UV-light dry-aging (UDA)]. Figure S4: Microbiome beta diversity measures on microbial community collected from cull cow beef loins (M. longissimus lumborum) aged with different aging methods [Wet aging (WA), Dry aging (DA), Dry aging in water-permeable bag (DWA) and UV-light dry-aging (UDA)], Table S1: Effect of different aging methods on free fatty acid profiles composition of cull cow beef loins (M. longissimus lumborum) after 28 days of aging; Table S2: Top 10 metabolites loading score from each principal component from cull cow beef loins (M. longissimus lumborum) after 28 days of aging using different aging methods Different aging treatments: Wet-aging (WA), Conventional dry-aging (DA), Dry-aging in water permeable bag (DWA) and UV-light dry-aging (UDA). Table S3: Potential microbial marker correlated with changes observed in cull cow beef loins (M. longissimus lumborum) after 28 days of aging using different aging methods identified through both LEFSE (*p* < 0.05, FDR < 0.05) and ANCOM (*p* < 0.05, FDR < 0.05) anal-yses. Different aging treatments: Wet-aging (WA), Conventional dry-aging (DA), Dry-aging in water permeable bag (DWA) and UV-light dry-aging (UDA). Table S4: Co-occurrence analysis results showing unique ASVs (genus level) and metabolites pairs identified from cull cow beef loins (M. longissimus lumborum) aged using the different aging methods for 28 days. (*p*-value < 0.05, Rho > 0.8). Different aging treat-ments: Wet-aging (WA), Conventional dry-aging (DA), Dry-aging in water permeable bag (DWA) and UV-light dry-aging (UDA).
