# Supplementary material for: Characterizing the Flavor Precursors and Liberation Mechanisms of Various Dry-Aging Methods in Cull Beef Loins Using Metabolomics and Microbiome Approaches
Source: Metabolites. 2022 May 24;12(6):472. doi: 10.3390/metabo12060472 (PMC9230681; doi:10.3390/metabo12060472)
Supplement: Supplementary file 1 [file metabolites-12-00472-s001.zip › metabolites-1710548-supplementary.pdf]

metabolites

MDPI

Supplementary Data

Metabolomics and Microbiome Approaches to Elucidate Flavor Precursor and Liberation Mechanisms Related to Dry-aged Cull Beef Loins

Derico Setyabrata [1], Kelly Vierck [2], Tessa R. Sheets [1], Jerrad F. Legako [2], Bruce R. Cooper [3], Timothy A. Johnson [1], and Yuan H. Brad Kim[1,*]

[1] Department of Animal Science, Purdue University, West Lafayette, IN 47907, U.S.A.; dsetyabr@purdue.edu / dericos@uark.edu (D.S.), sheets27@purdue.edu (T.R.S.), john2185@purdue.edu (T.A.J)
[2] Bindley Bioscience Center, Purdue University, West Lafayette, IN 47907, U.S.A.; brcooper@purdue.edu (B.R.C.)
[3] Department of Animal and Food Science, Texas Tech University, Lubbock, TX, 79409, USA; kelly.vierck@ttu.edu / vierck@uark.edu (K.V.), jerrad.legako@ttu.edu (J.F.L.)
* Correspondence: bradkim@purdue.edu; Tel.: +1-765-496-1631

Table S1. Effect of different aging methods on free fatty acid profiles composition of cull cow beef loins (M. *longissimus lumborum*) after 28 days of aging.

Fatty Acid (%FA/total FA)	Type	WA	DA	DWA	UDA	SEM	P-value
C10:0	SFA	0.045	0.043	0.047	0.048	0.003	0.6764
C12:0	SFA	0.056	0.052	0.059	0.060	0.003	0.3028
C13:1	MUFA	0.016[c]	0.048[b]	0.167[a]	0.153[ab]	0.039	0.0184
C14:0	SFA	2.643	2.434	2.790	2.824	0.160	0.2895
C14:1n5	MUFA	1.103	0.873	1.117	1.040	0.115	0.4254
C15:0	SFA	0.273[a]	0.263[a]	0.326[a]	0.182[b]	0.024	0.0009
C16:0	SFA	28.148	28.541	28.480	29.274	0.489	0.4313
C16:1trans	MUFA	0.017	0.036	0.031	0.024	0.008	0.3438
C16:1n7	MUFA	5.509	4.756	5.603	5.016	0.269	0.0982
C17:0	SFA	0.698	0.699	0.658	0.676	0.026	0.6366
C17:1	MUFA	0.790[a]	0.688[b]	0.710[b]	0.649[b]	0.027	0.0049
C18:0	SFA	11.083	12.056	11.137	11.899	0.377	0.1642
C18:1trans	MUFA	0.352	0.447	0.369	0.376	0.056	0.4618
C18:1n9	MUFA	42.591	42.012	40.852	40.480	0.615	0.0669
C18:1n7	MUFA	1.789	1.625	1.851	1.794	0.138	0.6879
C18:2trans	PUFA	0.061[bc]	0.128[b]	0.196[a]	0.051[c]	0.021	0.0021
C18:2n6	PUFA	2.820	3.225	3.373	3.189	0.236	0.3802
C18:3n3	PUFA	0.061[c]	0.069[ab]	0.062[bc]	0.070[a]	0.003	0.0371
C18:3n6	PUFA	0.258	0.245	0.255	0.242	0.011	0.6406
C19:0	SFA	0.119	0.102	0.106	0.098	0.006	0.1008
C19:1	MUFA	0.023	0.023	0.022	0.027	0.003	0.7092
C20:0	SFA	0.150	0.191	0.152	0.175	0.014	0.1581
C20:1n9	MUFA	0.072	0.071	0.060	0.063	0.005	0.2623
C20:1n11	MUFA	0.427	0.343	0.377	0.341	0.028	0.0621
C20:2	PUFA	0.034[ab]	0.025[b]	0.037[a]	0.037[a]	0.003	0.0294
C20:3n6	PUFA	0.094	0.107	0.122	0.132	0.015	0.3124
C20:4n6	PUFA	0.532	0.573	0.710	0.716	0.096	0.4286
C20:5	PUFA	0.030[b]	0.042[ab]	0.046[a]	0.058[a]	0.006	0.0136

Table S1. Continued

C22:3	PUFA	0.006	0.007	0.006	0.009	0.001	0.129
C22:4	PUFA	0.004	0.004	0.010	0.004	0.003	0.4551
C22:5n3	PUFA	0.122	0.150	0.180	0.166	0.021	0.2425
C22:6n3	PUFA	0.004	0.006	0.011	0.009	0.002	0.0759
C24:0	SFA	0.003	0.006	0.004	0.005	0.001	0.1229
C24:1n9	MUFA	0.107	0.119	0.138	0.139	0.020	0.6374
SFA%		44.143	43.599	43.611	45.235	0.741	0.3808
MUFA%		51.342	52.089	51.678	50.144	0.712	0.2858
PUFA%		4.515	4.312	4.711	4.656	0.354	0.5164
Total Free Fatty Acids Wet Basis (mg/g wet meat)		82.04	83.09	73.71	72.03	13.14	0.6804
Total Free Fatty Acids Dry Basis (mg/g dry meat)		260.26	208.29	213.35	154.21	38.62	0.0689

$^{a\text{-}c}$ Different superscript letters indicated a significant difference between the different aging methods (P<0.05)
Different aging treatments: Wet-aging (WA), Conventional dry-aging (DA), Dry-aging in water-permeable bag (DWA) and UV-light dry-aging (UDA)
SFA: Saturated fatty acid, MUFA: Monounsaturated fatty acid, PUFA: Polyunsaturated fatty acid
SEM: Standard Error of Means

Table S2. Top 10 metabolites loading score from each principal component from cull cow beef loins (M. *longissimus lumborum*) after 28 days of aging using different aging methods Different aging treatments: Wet-aging (WA), Conventional dry-aging (DA), Dry-aging in water permeable bag (DWA) and UV-light dry-aging (UDA)

Principal Component	Compound Name	Loading score (Absolute Value)
PC1	Ile-Ile	0.212
	Leu-Leu-Leu	0.194
	Lysophosphatidylethanolamine	0.193
	Lactoylglutathione	0.191
	Unknown3	0.185
	Unknown1	0.179
	Unknown4	0.176
	Proline	0.168
	Lysophosphatidylethanolamine	0.167
	Tetrahydrofurfuryl cinnamate	0.166
PC2	Glutathionyl acetate	0.246
	Asp-Cys	0.242
	Glutathionyl acetate	0.241
	Thioproline	0.240
	Unknown16	0.240
	Unknown14	0.226
	His-Glu	0.215
	Unknown5	0.184
	Phenylethyl glucopyranoside	0.180
	Gln-Gln	0.168
PC3	Unknown13	0.174
	Hyp-Hyp	0.161
	Tyromycic acid	0.152
	Histidine	0.152
	Linalooloxide apiosylglucoside	0.148
	Unknown12	0.147
	Coumaric acid	0.143
	His-Glu	0.138
	Demethoxymatteucinol	0.134
	Unknown2	0.131
PC4	Erysothiopine	0.301
	Coumaric acid	0.291
	Deoxyfructosyl Tyrosine	0.286
	Unknown9	0.251
	Unknown6	0.241
	Tyromycic acid	0.223
	Lysophosphatidylethanolamine	0.220
	Lysophosphatidylethanolamine	0.210

	Oxoursadienoate	0.198
	Unknown11	0.184
PC5	Acetyl-Lysine	0.316
	Unknown9	0.290
	Triethyl citrate	0.285
	Histidine	0.279
	Tetraethylene glycol	0.240
	Unknown11	0.225
	Trimethyloxazoline	0.215
	Unknown12	0.204
	Lactoylglutathione	0.192
	Val-Ser	0.187

Table S3. Potential microbial marker correlated with changes observed in cull cow beef loins (M. *longissimus lumborum*) after 28 days of aging using different aging methods identified through both LEFSE (P<0.05, FDR<0.05) and ANCOM (P<0.05, FDR<0.05) analyses. Different aging treatments: Wet-aging (WA), Conventional dry-aging (DA), Dry-aging in water permeable bag (DWA) and UV-light dry-aging (UDA)

ASVs	Enriched Treatment
Pseudomonas ASV1	DA
Unclassified Yersiniaceae ASV1	WA
Carnobacterium	WA
Unclassified Lactobacillales	WA
Brochothrix	WA

Table S4. Co-occurrence analysis results showing unique ASVs (genus level) and metabolites pairs identified from cull cow beef loins (M. longissimus lumborum) aged using the different aging methods for 28 days. (P-value <0.05, Rho > 0.8). Different aging treatments: Wet-aging (WA), Conventional dry-aging (DA), Dry-aging in water permeable bag (DWA) and UV-light dry-aging (UDA)

Treatment	Metabolites	ASV
WA	Acetyl-Lysine	*Acinetobacter* ASV1
	Deoxyfructosyl Tyrosine	*Carnobacterium, Lactobacillus, Psychromonas*
	Glutaminyl-Glutamine	*Aeromonas*
	Heptanethiol	*Aeromonas*
	Proline	*Flavobacterium* ASV2, *Massilia, Rothia, Shewanella,* Unclassified Gammaproteobacteria
	Tetraethylene glycol	*Acinetobacter* ASV1
	Triethyl citrate	*Carnobacterium, Lactobacillus, Psychromonas*
DA	Estradiol quinone	*Micrococcaceae, Mucor, Noccaea*
	Futoamide	*Micrococcaceae, Mucor, Noccaea*
	Glutaminyl-Glutamine	*Acinetobacter* ASV6, *Flavobacterium* ASV1
	Glutamyl-Histidine	*Actinomyces, Bacillus* ASV1, *Bacillus* ASV2, *Bacillus* ASV3, *Bifidobacterium, Chlorobi, Comamonadaceae* ASV1, *Comamonadaceae* ASV2, *Enterococcus* ASV2, *Enterococcus* ASV3, *Ferruginibacter, Geobacillus, Mesorhizobium, Metagenome* ASV1, *Metagenome* ASV2, *Microbacteriaceae, Proteiniphilum, Streptococcus* ASV2
	Linalooloxide apiosylglucoside	*Flavobacterium* ASV1, *Flavobacterium* ASV2,*Flavobacterium* ASV3, *Pedobacter* ASV2, *Staphylococcus, Vagococcus*
	Methyleneproline	*Acinetobacter* ASV6, *Flavobacterium* ASV1, *Pedobacter* ASV1
	Proline	*Actinomyces, Bacillus* ASV1, *Bacillus* ASV2, *Bacillus* ASV3, *Bifidobacterium, Chlorobi, Comamonadaceae* ASV1, *Comamonadaceae* ASV2, *Enterococcus* ASV2, *Enterococcus* ASV3, *Ferruginibacter, Geobacillus, Mesorhizobium, Metagenome* ASV1, *Metagenome* ASV2, *Microbacteriaceae, Proteiniphilum, Streptococcus* ASV2
	Phenylalanyl-Phenylalanine	*Micrococcaceae, Mucor, Noccaea*
	Seryl-Isoleucine	*Micrococcaceae, Mucor, Noccaea*
DWA	Heptanethiol	*Chryseobacterium* , Unclassified Enterobacteriaceae
	Hydroxyprolyl-Hydroxyproline	*Chryseobacterium* , Unclassified Enterobacteriaceae
	Oxoursadienoate	*Chryseobacterium* , Unclassified Enterobacteriaceae
	Tyromycic acid	*Chryseobacterium* , Unclassified Enterobacteriaceae
UDA	Glutaminyl-Glutamine	*Acinetobacter* ASV2, *Acinetobacter* ASV3, *Acinetobacter* ASV4, *Acinetobacter* ASV5, *Acinetobacter* ASV7, *Corynebacterium* ASV1, *Corynebacterium* ASV2, *Enterococcus* ASV1, *Granulicatella, Leuconostoc, Neisseria,* Unclassified Pasteurellaceae, *Phyllobacterium, Rothia, Streptococcus* ASV1, *Streptococcus* ASV3
	Lactoylglutathione	*Acinetobacter* ASV2, *Acinetobacter* ASV3, *Acinetobacter* ASV4, *Acinetobacter* ASV5, *Acinetobacter* ASV7, *Corynebacterium* ASV1, *Corynebacterium* ASV2, *Enterococcus* ASV1, *Granulicatella, Leuconostoc, Neisseria,* Unclassified Pasteurellaceae, *Phyllobacterium, Rothia, Streptococcus* ASV1, *Streptococcus* ASV3

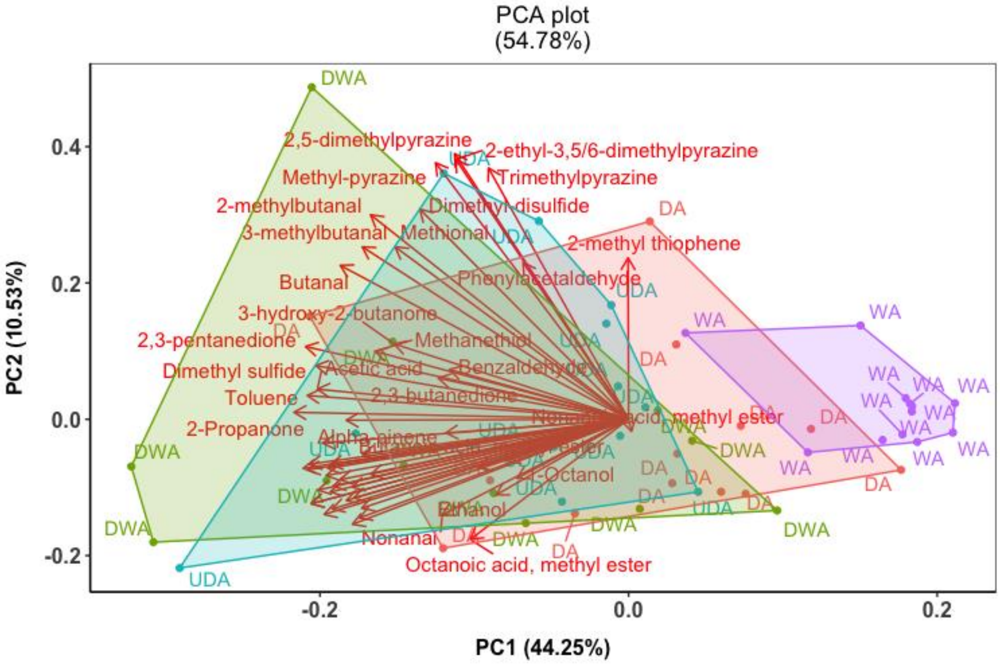

Figure S1. Principal component analysis (PCA) biplot of volatile compounds from cull cow beef loins (M. *longissimus lumborum*) aged with different aging methods [Wet aging (WA), Dry aging (DA), Dry aging in water-permeable bag (DWA) and UV-light dry-aging (UDA)].

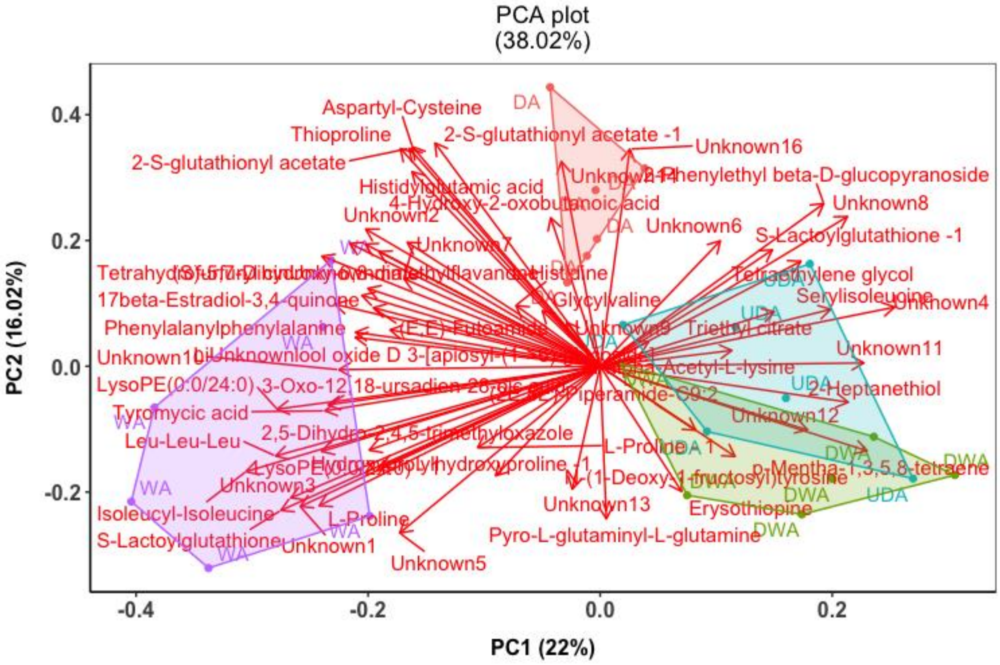

Figure S2. Principal component analysis (PCA) biplot of metabolites from cull cow beef loins (M. *longissimus lumborum*) aged with different aging methods [Wet aging (WA), Dry aging (DA), Dry aging in water-permeable bag (DWA) and UV-light dry-aging (UDA)].

(a) Chao1 Richness Index

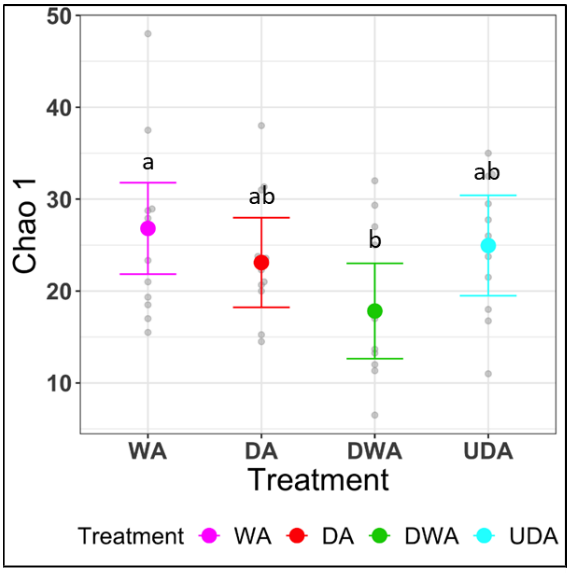

	Effect	P-value
Chao1	Treatment	0.0284
	Source	0.3039
	Treatment x Source	0.0549

(b) Pielou Evenness Index

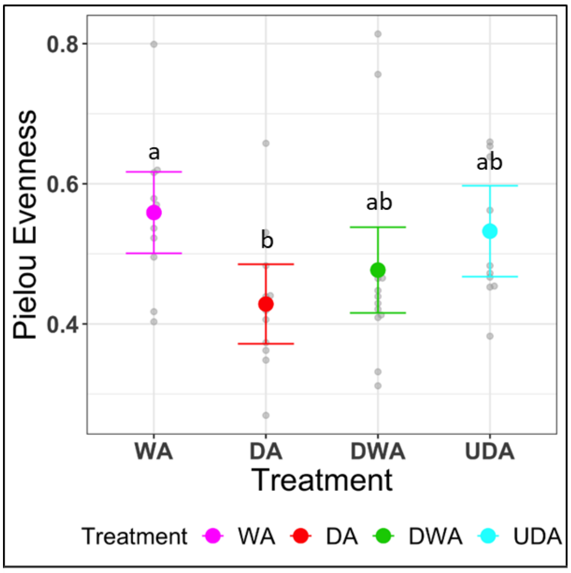

	Effect	P-value
Pielou Evenness	Treatment	0.0099
	Source	0.8893
	Treatment x Source	0.4027

(c) Faith Diversity Index

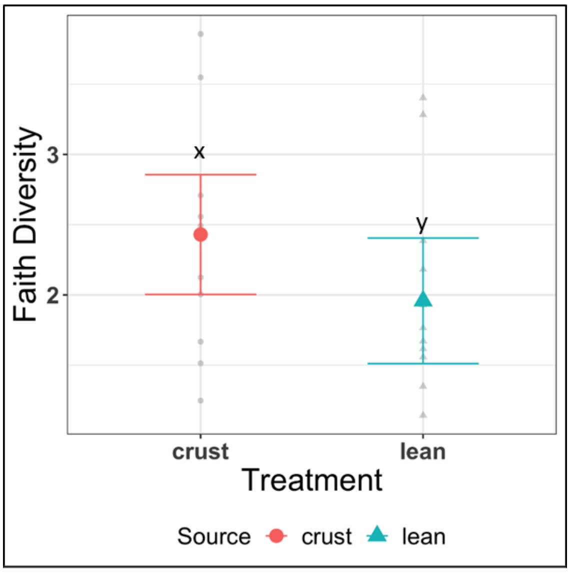

	Effect	P-value
Faith Diversity	Treatment	0.1969
	Source	0.0117
	Treatment x Source	0.1109

Figure S3. Microbiome alpha diversity index on microbial community collected from cull cow beef loins (M. *longissimus lumborum*) aged with different aging methods [Wet aging (WA), Dry aging (DA), Dry aging in water-permeable bag (DWA) and UV-light dry-aging (UDA)].

(a) Bray-Curtis Dissimilarity (b) Weighted Unifrac Distance

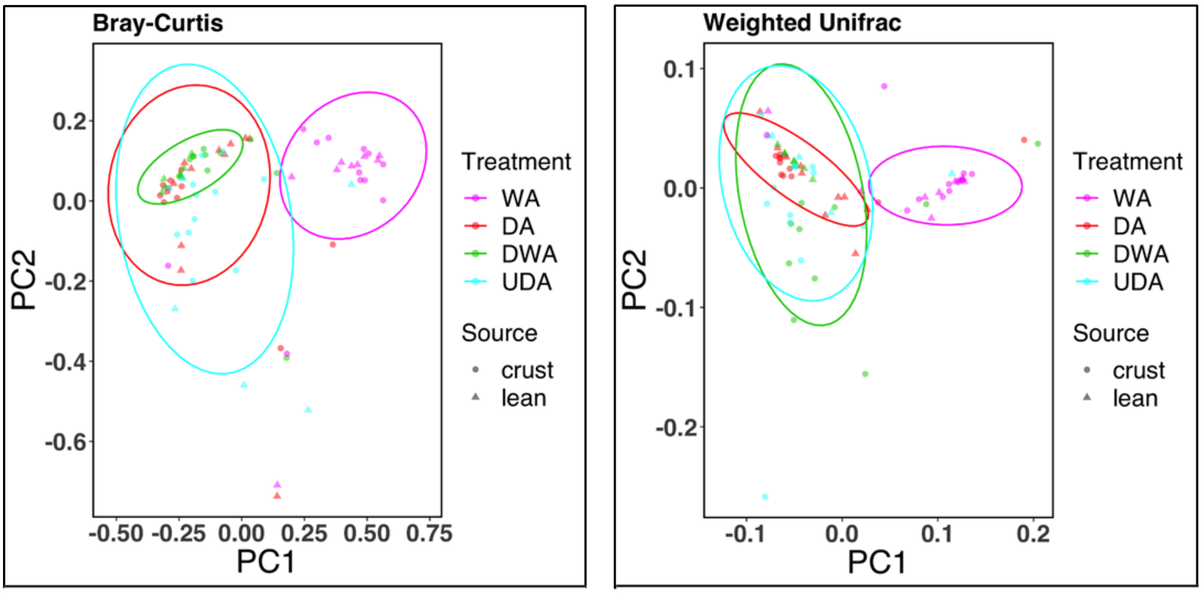

	Effect	P-value
Homogeneity Test	Treatment x Source	0.467
PERMANOVA Test	Treatment	0.001
	Source	0.092
	Treatment x Source	0.003

	Effect	P-value
Homogeneity Test	Treatment x Source	0.059
PERMANOVA Test	Treatment	0.001
	Source	0.024
	Treatment x Source	0.478

Figure S4. Microbiome beta diversity measures on microbial community collected from cull cow beef loins (M. longissimus lumborum) aged with different aging methods [Wet aging (WA), Dry aging (DA), Dry aging in water-permeable bag (DWA) and UV-light dry-aging (UDA)].
